# Identifying the Growth Modulon of *Corynebacterium glutamicum*

**DOI:** 10.3389/fmicb.2019.00974

**Published:** 2019-05-08

**Authors:** Thorsten Haas, Michaela Graf, Alexander Nieß, Tobias Busche, Jörn Kalinowski, Bastian Blombach, Ralf Takors

**Affiliations:** ^1^Institute of Biochemical Engineering, University of Stuttgart, Stuttgart, Germany; ^2^Center for Biotechnology (CeBiTec), Bielefeld University, Bielefeld, Germany; ^3^Institute for Biology-Microbiology, Freie Universität Berlin, Berlin, Germany; ^4^Microbial Biotechnology, Campus Straubing for Biotechnology and Sustainability, Technical University of Munich, Straubing, Germany

**Keywords:** *Corynebacterium glutamicum*, growth rate, growth modulon, transcript analysis, gene regulatory network, growth rate transition

## Abstract

The growth rate (μ) of industrially relevant microbes, such as *Corynebacterium glutamicum*, is a fundamental property that indicates its production capacity. Therefore, understanding the mechanism underlying the growth rate is imperative for improving productivity and performance through metabolic engineering. Despite recent progress in the understanding of global regulatory interactions, knowledge of mechanisms directing cell growth remains fragmented and incomplete. The current study investigated RNA-Seq data of three growth rate transitions, induced by different pre-culture conditions, in order to identify transcriptomic changes corresponding to increasing growth rates. These transitions took place in minimal medium and ranged from 0.02 to 0.4 h^-1^ μ. This study enabled the identification of 447 genes as components of the growth modulon. Enrichment of genes within the growth modulon revealed 10 regulons exhibiting a significant effect over growth rate transition. In summary, central metabolism was observed to be regulated by a combination of metabolic and transcriptional activities orchestrating control over glycolysis, pentose phosphate pathway, and the tricarboxylic acid cycle. Additionally, major responses to changes in the growth rate were linked to iron uptake and carbon metabolism. In particular, genes encoding glycolytic enzymes and the glucose uptake system showed a positive correlation with the growth rate.

## Introduction

The industrial potential of *Corynebacterium glutamicum* has attracted the attention of both researchers as well as industry experts, who are engaged in an attempt to clarify and improve its characteristics. *C. glutamicum*, a non-pathogenic, small rod-shaped, Gram-positive, soil bacterium, was discovered in 1957 (Kinoshita et al., 1957) and quickly utilized as a natural producer of L-glutamate (Hermann, 2003) and L-lysine (Becker et al., 2007; Takeno et al., 2010). Since then, it has been established as a prime producer of amino acids, including L-ornithine (Hadiati et al., 2014), L-arginine (Ikeda et al., 2009; Lubitz et al., 2016), L-valine (Oldiges et al., 2014), and L-histidine (Cheng et al., 2013; Kulis-Horn et al., 2014). *C. glutamicum* has several advantages as a production host, including great robustness to process deviations, excellent culture characteristics that are stable up to high cell densities, genetic stability, and broad utilization of carbon sources (Lee et al., 2016). Due to these advantages, many studies were conducted on *C. glutamicum* with the objective of expanding its scope of production toward producing other substances beyond amino acids including organic acids, alcohols, and polymers (Becker and Wittmann, 2015). Sequencing of the whole genome of *C. glutamicum* ATCC13032 strain (Ikeda and Nakagawa, 2003; Kalinowski et al., 2003) has been a major facilitator of this expansion. Whole genome sequencing, in combination with recent advances in high-throughput genomic analysis (Pfeifer-Sancar et al., 2013), has opened up new avenues for targeting molecular pathways of the strain via metabolic engineering. Recently *C. glutamicum* has gained recognition for its potential to host recombinant protein expression, largely due to advantages such as a lack of endotoxins, ability to secrete heterologous proteins, and high efficacy as a production host (Liu et al., 2016; Freudl, 2017).

However, a disadvantage of *C. glutamicum* is that it exhibits lower growth rates compared to some of its industrial competitors. The maximum growth rate reported for *C. glutamicum* ATCC13032 in minimal medium containing glucose under highly diluted conditions is 0.62 ± 0.02 h^-1^ (Grünberger et al., 2013). Under industrially relevant conditions, performance is substantially lower, with reported growth rates of 0.4–0.45 h^-1^ (Grünberger et al., 2013; Unthan et al., 2014). Because the growth rate is an industrially important factor that has a strong effect on the space-time yield of a process, a multitude of studies have been conducted to improve *C. glutamicum* in this regard (Yamamoto et al., 2012; Seibold and Eikmanns, 2013). Adaptive laboratory evolution (ALE) experiments have proven to be the most successful approach for achieving higher growth rates. In these experiments, selection pressure is applied over an extended processing time, using serial dilutions or continuous culture methods, to select for randomly occurring mutations that increase desired properties (Dragosits and Mattanovich, 2013). Using this approach, Pfeifer et al. (2017) achieved an increase in the growth rate of 28.8 ± 1.9% and Wang et al. (2018), an increase of 42%. These authors used systems biological tools to clarify causative mutations.

Many regulatory relationships have been identified since the sequencing of the strain (Wennerhold and Bott, 2006; Engels and Wendisch, 2007; Auchter et al., 2011; Pátek and Nešvera, 2011; Neshat et al., 2014). Current information regarding regulatory interactions, deposited in the CoryneRegNet database (Pauling et al., 2012), has led to the identification of complex regulatory networks (Shah et al., 2018). Although much progress has been made in the elucidation of regulatory interactions (Schröder and Tauch, 2010; Nomura, 2014), mechanisms underlying growth rate transition remain unclear. This slows targeted improvement of the growth performance of this strain, leaving unguided evolutionary approaches as the most successful tools thus far.

In order to increase the knowledge base associated with regulatory responses to changes in the growth rate under dynamic conditions of standard processes, the growth modulon of *C. glutamicum* was identified. For this purpose, transcriptome samples taken during growth rate transitions were evaluated. These transitions followed three different pre-culture conditions which were compared to assess the effect of substrate limitation during previous cultivations. Basically, growth rate and growth phase transitions are reflected within the gathered raw data. The latter could be extracted by analyzing the shift from lag to exponential growth phase, whereas the former was covered by studying different growth rates. However, the focus of the study is set on growth rate transitions exploiting the richness of experimental data obtained while investigating different growth rates. Based on our results key regulators and pathways involved in the transition were identified. Such knowledge may improve the understanding of growth rate regulation in *C. glutamicum*, enabling identification of key targets that may be manipulated to develop strains with increased growth rates and space-time yields. This may ultimately lead to knowledge-based growth optimization of *C. glutamicum* strains, thus increasing their industrial competitiveness.

## Materials and Methods

### Pre-culture

*Corynebacterium glutamicum* ATCC 13032 wild type (WT) cells, from glycerol stocks, were plated on 2 × tryptone-yeast extract (2 × TY) medium (Sambrook, 2001) agar plates and incubated for 60 h at 30°C. Glass reaction tubes with 5 mL of 2 × TY medium were inoculated with colonies from these plates and then incubated for 8 h at 30°C on a bench-top rotary shaker (Infors HT, Bottmingen, Switzerland) at 120 rpm.

For carbon limitation, the contents of six such glass reaction tubes were used to inoculate six baffled 500 mL shaking flasks, each containing 50 mL of modified CGXII minimal medium (Buchholz et al., 2014) supplemented with 1% (w/v) glucose and 30 mg L^-1^ protocatechuic acid (PCA) and cells were allowed to grow until the limiting substrate was fully consumed. The carbon-limited pre-culture was harvested 60 h after inoculation. For phosphate limitation, the content of one glass reaction tube was used to inoculate a 3 L baffled shaking flask with 300 mL of modified CGXII minimal medium, in which the concentrations of K_2_HPO_4_ and KH_2_PO_4_ were reduced to 0.01 g L^-1^ each. Cells were allowed to grow until available phosphate was consumed and resulting phosphate-limited pre-culture was harvested 84 h following inoculation. For nitrogen limitation, the content of one glass reaction tube was used to inoculate 300 mL of modified CGXII minimal medium without urea in a 3 L baffled shaking flask. 3-Morpholinopropane-1-sulfonic acid (MOPS) buffer concentration was doubled to 42 g L^-1^, and (NH_4_)_2_SO_4_ concentration was reduced to 0.1 g L^-1^. Cells were allowed to grow until available nitrogen was consumed and resulting nitrogen-limited pre-culture was harvested 48 h after inoculation.

### Bioreactor Cultivations

Bioreactor cultivations were carried out in triplicate in a 3 L KLF steel-tank bioreactor (Bioengineering, Wald, Switzerland). All bioreactor cultivations were performed with an initial volume of 1.7 L of CGXII minimal medium supplemented with 5% of glucose. Harvested pre-cultures were centrifuged for 10 min at 7000 × *g* and resuspended in sterile 0.9% (w/v) saline. The medium in the bioreactor was inoculated with a starting OD_600nm_ of 5 to improve cell dry weight (CDW) determination accuracy and reduce the volume needed for transcript samples. Temperature was maintained at 30°C, while pH was kept constant at 7.4 by the addition of 25% (v/v) NH_4_OH. The aeration rate (V_Air_) was 1 L min^-1^ and the pressure in the reactor was maintained at 1.5 bar. The stirrer speed of the six bladed Rushton impeller was automatically adjusted, so that dissolved oxygen levels in the reactor did not drop below 30% of the physical maximum. Foaming was counteracted by manually adding the antifoaming agent Struktol J 647 (Schill + Seilacher, Hamburg, Germany). Exhaust gas analysis of O_2_ and CO_2_ fractions was performed with infrared gas analyzers BCPO2 and BCPCO2 (BlueSens, Herten, Germany).

### Biomass Concentration

Cell dry weight was measured gravimetrically in technical quadruplets. One milliliter of cell suspension was centrifuged at 20000 × *g* and 4°C for 2 min, washed twice with deionized water and dried in glass vials for at least 24 h at 105°C in a convection oven (Heraeus, Hanau, Germany). Following removal from the oven, the glass vials were cooled in a desiccator for a minimum of 3 h and closed in an airtight manner with a plastic lid. The CDW was measured using an XPE micro analytical balance (Mettler Toledo GmbH, Albstadt, Germany).

### Glucose Concentrations

The glucose concentration of cell free supernatant was determined via enzymatic assay D-Glucose Cat. No. 10716251035 according to the instructions of the manufacturer (R-biopharma, Darmstadt, Germany).

### Viability

Viability of each cell suspension was determined by the number of colony-forming units in biological triplicates. The cell suspension was diluted with sterile 0.9% saline, to ensure between 100 and 200 colony-forming units were present per mL of cell suspension. One hundred microliters of the diluted cell suspension were then plated on 2 × TY medium agar plates and inoculated at 30°C. The number of colonies was counted after 48 h. One hundred percent viability was defined as the number of viable colonies per volume of a sample taken during mid-exponential growth phase. Percentage viability for stationary cultures was determined based on the above standard.

### Determination of the Differential Growth Rate

The viability adjusted differential growth rate μ_diff,*t*_ was determined for each sample using Eq. 1.

(1) $$\mu_{\mathrm{diff},\;t}=\ln\;\left(\frac{c_{x,t}-c_{x,0}*\frac{\upsilon_{0}}{100}}{c_{x,t-1}-c_{x,0}*\frac{\upsilon_{0}}{100}}\right)$$

where υ_0_ is percent viability of the cells at inoculation, and *c_x_* is biomass concentration at time *t*.

To estimate the differential growth rate between the CDW samples, a linear increase of μ_diff_ between time points was assumed. Based on this assumption, a simple linear regression model was fitted between each neighboring point μ_diff,*t*_ and μ_diff,*t*-1_ to estimate the growth rate between the points.

### Transcriptome Cell Sampling

Transcript samples were prepared via fast centrifugation. Two milliliters of each sample were centrifuged at 20000 *g* for 30 s at 4°C, the supernatant was removed, and the sample was immediately flash frozen in liquid nitrogen. During the impaired growth phase, transcript samples were drawn every hour. During growth rate acceleration, transcript samples were drawn at 20 min intervals. Following estimation of the differential growth rate for each transcript sample using the regression model, samples that fit the target growth rates of 0, 0.1, 0.2, 0.3, and 0.4 h^-1^ as closely as possible, were picked for transcript measurement. The replicates, C2-0.1 and N3-0.2, were rejected in a quality assessment, and thus, only two biological replicates were available for analysis.

### RNA Isolation

Total RNA was isolated from biological replicates using RNeasy Mini Kit and a DNase Kit (both from Qiagen) as described previously (Busche et al., 2012). Initially, RNA quality was checked using Trinean Xpose (Gentbrugge, Belgium) and Agilent RNA Nano 6000 kit on Agilent 2100 Bioanalyzer (Agilent Technologies). Samples contaminated with DNA were treated with DNase (Qiagen), cleaned as described above, and rechecked via Xpose and Agilent Bioanalyzer. Finally, RNA with an RNA Integrity Number (RIN) >9 and an rRNA Ratio [23S/16S] >1.5, was confirmed as free of DNA.

### Whole Transcriptome Sequencing

High-quality total RNA aliquots of the biological replicates were rRNA-depleted using the Illumina Ribo-Zero rRNA Removal Kit for Bacteria. Successful rRNA depletion was confirmed using Agilent RNA Pico 6000 kit on Agilent 2100 Bioanalyzer (Agilent Technologies). The TruSeq Stranded mRNA Library Prep Kit from Illumina, starting with fragment, prime, and finish was used to prepare the cDNA libraries. The cDNA libraries were then sequenced in paired-end mode on an Illumina HiSeq 1500 system with 50 respectively 70 bases read length, and Illumina MiSeq system with 75 bases read length. The raw sequencing data have been deposited in the ArrayExpress database at EMBL-EBI^1^ under accession number E-MTAB-7436.

### Read Mapping and Raw Read Count Calculation

Quality filtered and trimmed sequence reads (trimmomatic v0.36: LEADING:3 TRAILING:3 SLIDINGWINDOW:4:15 MINLEN:36) (Bolger et al., 2014) of all replicates were mapped to the reference genome *Corynebacterium glutamicum ATCC13032 strain IBVT* using bowtie2 v2.2.7 with default settings for paired-end read mapping. Gene annotation for this strain is attached in the Supplementary Material (Supplementary Data Sheet 1).

Raw read count calculations per CDS and visualization was performed using the short-read mapping analysis platform, ReadXplorer 2.2.3 (Hilker et al., 2016).

### Data Analysis

Data analysis was performed using R 3.5.0. Raw read counts were normalized using the Relative Log Expression function of the DESeq2 (Love et al., 2014) package. The Poisson dissimilarity matrix was calculated using the PoiClaClu (Witten, 2011) package. Clustering and visualization of the dissimilarity matrix was achieved using pheatmap. Differential gene expression for each condition was determined separately using the maSigPro (Nueda et al., 2014) package in “generalized linear model” mode. A log_2_-fold change cutoff of 0.73, 0.73, and 0.6 (lowered due to the lack of a sample for the lowest growth rate) was used for phosphate, nitrogen, and carbon limitation samples, respectively. Identification of expression profile similarities for each gene was conducted using maSigPro (Nueda et al., 2014). The Pearson correlation matrix, on which clustering of expression profiles was based, was calculated using the Cor function. Clustering of the resultant matrix was performed using the hclust function. KEGG enrichment was performed using the Cluster Profiler package (Yu et al., 2012). Overrepresentation analysis was performed using the phyper function of the stats packageand was solely based on experimentally verified regulatory interactions of *Corynebacterium glutamicum* ATCC13032 as curated in the database^2^. For every *p-*value presented, the false discovery rate (FDR) was controlled using the Benjamini–Hochberg method through the function *p.adjust* in R. If not stated otherwise statistical significance was set at *adjusted p-*value <0.05. The network was visualized using Cytoscape (Shannon et al., 2003). All *other* visualizations were performed in R using ggplot2 (Wickham, 2016)

## Results

### Monitoring Transcription Dynamics During Growth Acceleration

Transcriptional regulation of the growth rate of *C. glutamicum* ATCC13032 was studied using a data-series approach. Since the strain did not show a lag phase when transferred from complex to fresh minimal medium, lag phases were induced by exposing the strain to long-term stationary periods in preceding cultures. Three different pre-culture conditions, phosphate, nitrogen, and carbon limitation, were imposed to induce individual prolonged stationary periods. Accordingly, distinct phases of growth acceleration were observed following subsequent transitions to batch cultivations with fresh minimal medium. Furthermore, preliminary studies (data not shown) indicated that cell viability was reduced following exposure to such stationary conditions. In order to prevent biased growth rate measurements, differential growth rates were adjusted by only considering viable cell populations. Consequently, initial batch biomass values were corrected using measured viabilities of 60 ± 5% for phosphate limitation, 96 ± 4% for nitrogen limitation and 88 ± 4% for carbon limitation.

**FIGURE 1:**
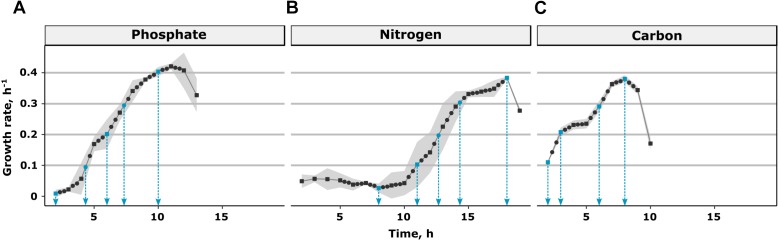
Profiles of growth rate transitions in fresh CGXII minimal medium after a **(A)** phosphate, **(B)** nitrogen, and **(C)** carbon limited pre-culture. All dots represent transcript samples taken. Differential growth rates (squares) are calculated based on biomass measurements. The black line represents the regression of growth rates. Samples for which the transcriptome has been measured are indicated with a blue arrow.

The growth profiles after inoculation of the fresh minimal medium with the stationary cells exhibited a clear growth rate transition following a lag phase of varying length dependent on the pre-culture condition (Figure 1). The longest lag phase of 10 h was observed for nitrogen limitation, while it had the shortest pre-culture of 48 h. The pre-cultures of the carbon limitation were 12 h longer than that of nitrogen limitation while those of the phosphate limitation were 36 h longer than those of nitrogen limitation. Still, these longer stationary phases did result in substantially shorter lag phases. In case of carbon limitation, the initial lag phase was too short be observable. Therefore, no samples at a growth rate of 0 h^-1^ could be obtained after carbon limitation. The initial lag phase was followed by a phase of increasing differential μ which lasted over several hours. This phase of growth acceleration was similar in length for all three conditions and the maximum growth rate reached was 0.4 ± 0.02 h^-1^. Transcript measurement samples (indicated by blue arrows), were taken at the lowest growth rate (defined as μ = 0 h^-1^) and at μ = 0.1, 0.2, 0.3, and 0.4 h^-1^. This led to a total of three datasets, each with three biological replicates, containing transcriptional regulation of growth rate transition within minimal medium, and a range of growth rate changes from μ = 0 to 0.4 h^-1^.

### Clustering Transcriptional Data

**FIGURE 2:**
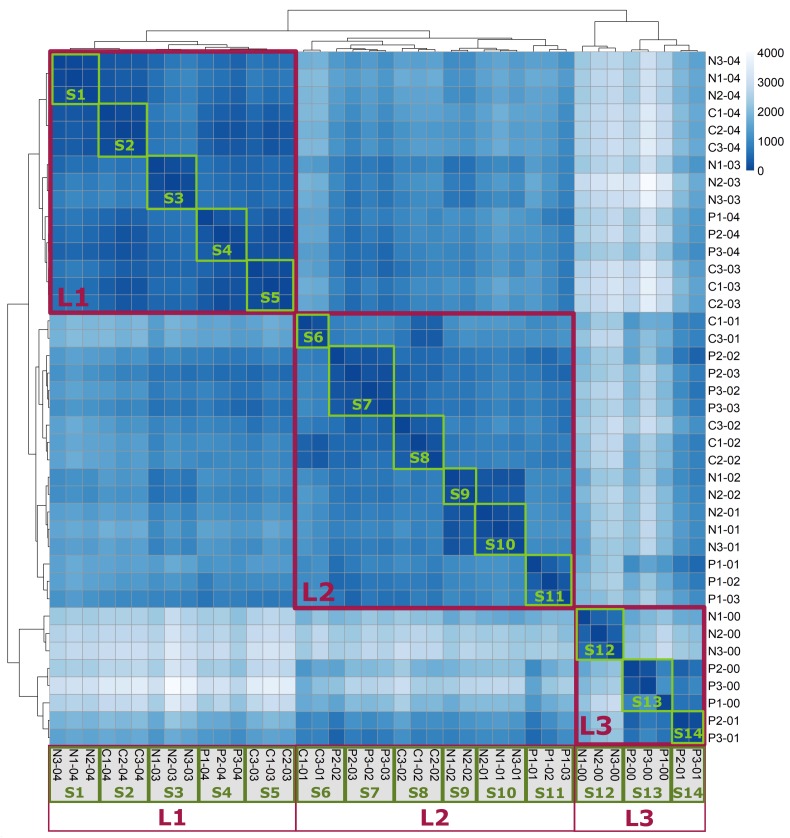
Heatmap of Poisson dissimilarity matrix between samples. The shade indicates similarity between samples, with a darker hue representing higher similarity. The sample name consists of previously limiting condition, number of the biological replicate, and differential growth rate at sampling. Clusters L1–L3 (red frames) consist of samples taken during similar growth rates. Clusters S1–S14 (green frames) mainly consist of biological replicates.

Hierarchical clustering of all normalized raw reads counts was performed according to a Poisson dissimilarity matrix. The heatmap representing this clustering exhibited three major clusters, indicated by red frames (Figure 2). Cluster L1 exclusively contained samples taken during the highest growth rates of 0.3 and 0.4 h^-1^, cluster L2 predominantly comprised growth rates of 0.1 and 0.2 h^-1^, while cluster L3 consisted of samples with the lowest growth rates. Notably, the composition of these clusters was dependent on growth rate, and independent of previous limiting conditions. On a smaller scale, clustering also identified a majority of biological replicates obtained under the same condition and at the same growth rate (green frames, S1–S14).

### Differential Gene Expression Analysis and Pathway Enrichment

**FIGURE 3:**
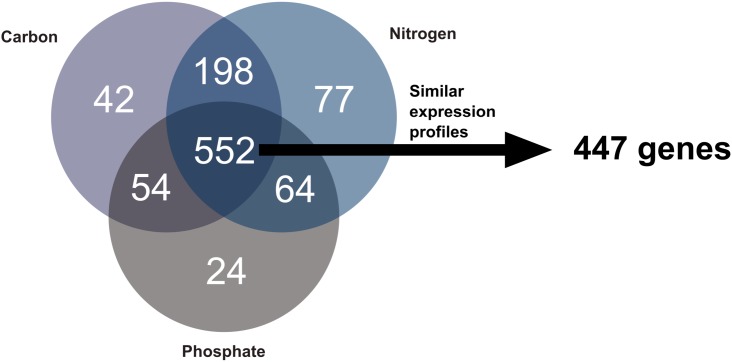
Comparison of DEGs during growth rate transitions after different pre-culture conditions. Gene expression profiles of the 552 genes, which are DE in all three conditions, were compared using a cubic regression model, and genes exhibiting similar profiles in all three conditions were identified. This enabled the identification of the growth modulon comprising 447 genes.

Three groups of differentially expressed genes (DEGs) containing similar gene numbers with 673, 861, and 801 genes were identified for phosphate, nitrogen, and carbon limitation, respectively. These groups overlapped considerably, sharing a total of 552 genes between conditions (Figure 3). This amounted to 80% of all differentially expressed (DE) genes, following phosphate limitation, 66% following carbon limitation and 62% following nitrogen limitation. The overlap between DEGs in nitrogen and carbon limitation was more than three times larger than that between phosphate and the other two conditions. Only a relatively small number of genes identified as DEGs was particularly observed under a single condition. A further filter was applied to these 552 genes so that only genes showing similar expression profiles under all three conditions were included in the modulon of growth rate transition. This reduced the number of genes to a total of 447 (Supplementary Data Sheet 2). These genes formed the “growth modulon,” which was free from any influence of previous limitations.

**FIGURE 4:**
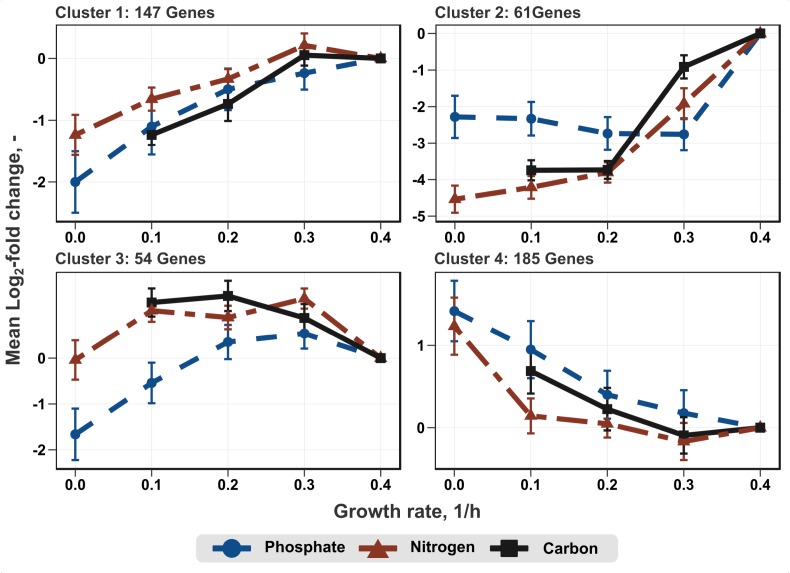
Average log_2_-fold change of genes of the growth modulon after clustering. The clustering was performed according to the similarity of gene expression profiles using a Pearson correlation matrix. The sample taken at μ = 0.4 h^-1^ was used as reference for log_2_-fold change calculation.

**FIGURE 5:**
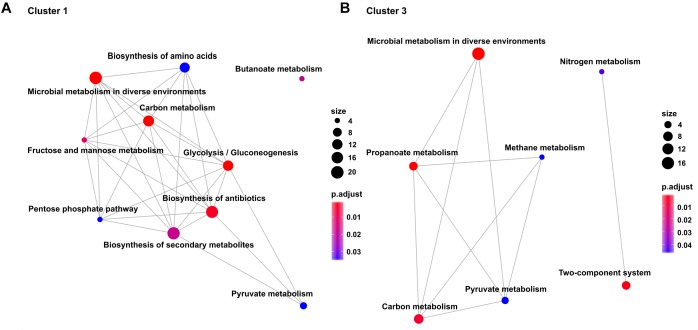
Enrichment maps showing the significantly over-represented KEGG Pathways (*p* < 0.025) within **(A)** Cluster 1 and **(B)** Cluster 3. Size of the dots represents total amount of genes within the category and color indicates the Benjamini–Hochberg adjusted *p*-value. The lines connecting categories represent an overlap between genes within the categories.

Transcription profiles of the growth modulon used for hierarchical clustering based on a Poisson correlation matrix are shown (Figure 4). As a result, four distinct clusters containing similar gene expression tendencies covering all growth rates and all conditions were identified. Further separation of the clusters did not yield substantially different profiles. Furthermore, KEGG enrichment analysis was performed for the genes within these clusters to elucidate the general functions of genes within each cluster (Figure 5; clusters 1 and 3). Cluster 1 consisted of genes showing a steady increase in expression levels over the growth rate. The average increase in gene expression was between a log_2_-fold change of 1 to 2. KEGG enrichment analysis for this cluster mainly revealed genes associated with carbon metabolism. In particular, genes encoding glycolysis exhibited this expression trend. Cluster 2 consisted of genes which do not show a sizable change in expression level at low growth rates but show the strongest average increase of all genes during acceleration from 0.2 to 0.4 h^-1^. KEGG enrichment analysis indicated that solely ABC transporters, such as genes encoding proteins involved in iron uptake and metabolic functions showed this trend. Therefore, the enrichment map of this cluster is not shown in Figure 5. Cluster 3 showed an expression profile similar to cluster 1 during transition at low growth rates, but exhibited decreasing expression levels from μ = 0.3 to 0.4 h^-1^. Once more, genes encoding proteins involved in carbon metabolism were assigned to this cluster. Contrary to the general trends seen in clusters 1–3, cluster 4 contained genes with decreasing expression levels. Interestingly, no KEGG pathways could be assigned to cluster 4.

### Deciphering the Gene Regulatory Network (GRN)

**FIGURE 6:**
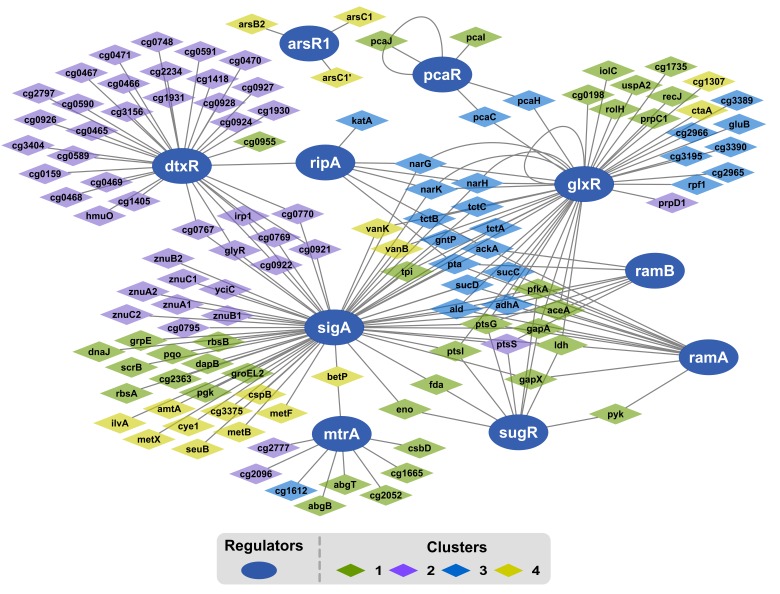
Network map of over-represented regulons within the growth modulon. The regulons have been identified using a hypergeometric test with FDR controlled via Benjamini–Hochberg (*p*-value <0.05). Regulators are represented as dark blue ellipses. Diamonds represent genes within the over-represented regulon. The color of the diamonds indicates the cluster assigned to the gene according to its expression profile. The edges between nodes indicate a regulatory interaction between the gene and the regulator.

Because gene expression of a regulon mirrors the activity of its regulator, DEG levels were analyzed to deduce the dynamics of correlated regulator genes. Of the 447 genes comprising the growth modulon, 28% may be assigned to at least one overrepresented regulon using prior knowledge of gene regulatory interactions curated in CoryneRegNet (Pauling et al., 2012). Ten regulators were statistically identified as overrepresented (Figure 6 and Table 1). At the center of the GRN was the housekeeping sigma factor SigA. Genes identified in the SigA regulon are a part of all expression profile clusters, as indicated via the color of the gene nodes. On the right side of the network, a highly interconnected cluster of regulators, consisting of the GlxR, SugR, RamA, and RamB regulons and their regulated genes, was present. Nearly all genes within this subnetwork were in either cluster 1 or cluster 3. Their expression levels correlated positively with growth. The iron response regulator DtxR was also identified, and genes in its regulon were overwhelmingly localized in cluster 2. Interestingly, the largest total log_2_-fold change was found for this regulon during the transition from μ = 0.2 to 0.4 h^-1^. Cellular membrane and stress response genes associated with the regulator MtrA were also identified and DEGs of the MtrA regulon mostly belonged to clusters 1 and 2. Two regulators unrelated to SigA, PcaR regulating one branch of the beta-ketoadipate pathway and ArsR1, were identified as overexpressed. Regulons of PcaR belonged to clusters 1 and 3 and those of ArsR1 belonged to cluster 4.

**Table 1:** Overview of significantly enriched regulons based on the number of genes, which are part of the growth modulon.

Regulator	Number of genes in the μ-modulon	Total number of genes in the regulon	Number of genes in the μ-modulon				*p.adj.*
				
			Cluster 1	Cluster 2	Cluster 3	Cluster 4	
DtxR	34	63	1	33	0	0	1.94E-15
SigA	62	250	20	16	14	12	1.45E-11
GlxR	43	180	14	1	24	4	2.43E-06
SugR	9	14	8	1	0	0	1.09E-05
RamB	7	17	2	1	4	0	0.007
RamA	13	45	7	0	6	0	0.009
ArsR1	3	5	0	0	0	3	0.010
RipA	6	15	0	0	6	0	0.011
PcaR	5	12	3	0	2	0	0.013
MtrA	9	31	5	2	1	1	0.023


*The p-value was adjusted for multiple testing using the Benjamini-Hochberg method, and statistical significance was expressed as *p.adj*. < 0.05.*

**FIGURE 7:**
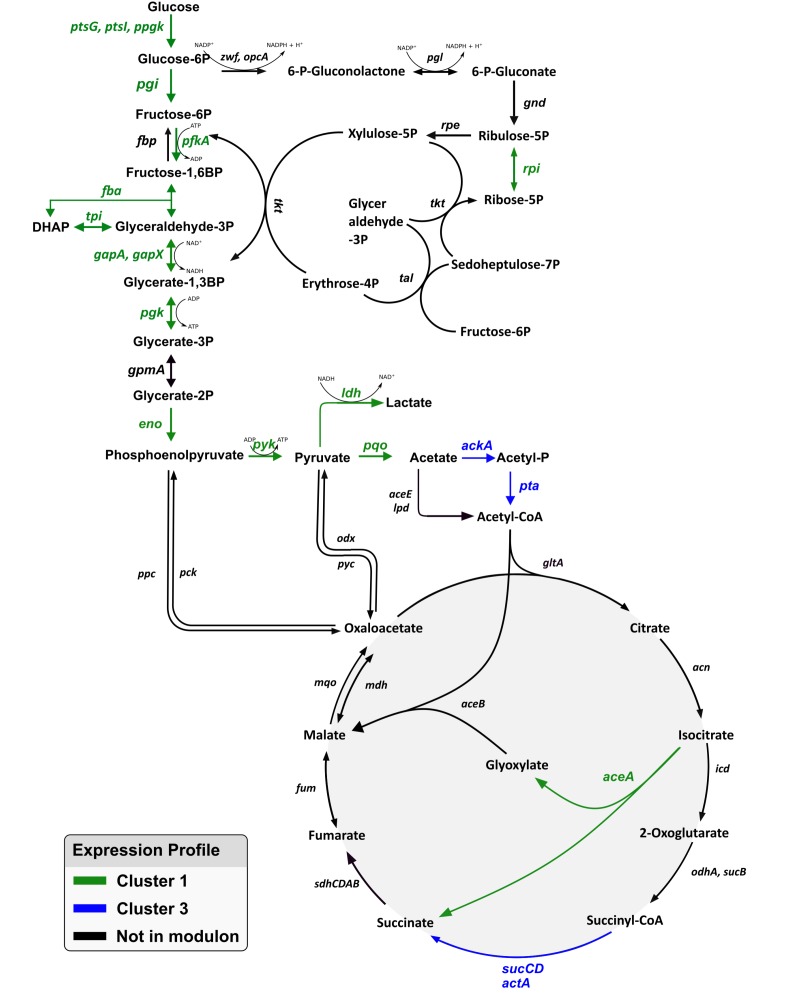
Representation of the genes in glycolysis, pentose phosphate pathway, and the TCA cycle. Genes identified as part of the growth modulon are highlighted in green and blue according to their gene expression profile. No genes of these pathways were found to exhibit the expression profiles of cluster 2 or 4.

Next, GRN findings were mapped for glycolysis, the pentose phosphate pathway (PPP) and the tricarboxylic acid cycle (TCA cycle) (Figure 7). In glycolysis, almost all genes were found to be members of the “growth modulon,” with the single exception of *gpmA* (cg0482). By contrast, the PPP comprised only of a single gene, namely *rpi* (cg2658), as a component of the “growth modulon.” In the TCA cycle only three genes, *aceA* (cg2658), *sucCD* (cg2037, cg 2038), and *actA* (cg2840), all of which encode enzymes facilitating reactions leading to succinate, were part of the modulon.

## Discussion

### Accounting for Viability Losses During the Preceding Stationary Phase

Cell viability decreases during periods of starvation (Han et al., 2007). This must be taken into account as calculation of differential μ is based on CDW measurements, which do not distinguish between viable and dead cells. Therefore, CDW values had to be corrected using initial viability of the culture at inoculation. Viability following phosphate limited pre-culture was determined to be the lowest of all viabilities measured at the end of pre-cultures. This reflects that cells experienced the longest stationary phase during phosphate limitation and highlights the importance of phosphate in cell survival (Rao and Kornberg, 1996). Interestingly, nitrogen starvation initiated the longest lag phase, even though it had the shortest stationary phase and the highest viability. Apparently, nitrogen starvation caused depletion of intracellular N-sources, such as amino acid pools. The shortage not only limited refueling of amino acids for biomass synthesis but also hindered the supply of proper tRNAs and ribosomes for translating mRNAs into proteins. Accordingly, cells are likely to be limited in cell growth due to metabolic and translational bottlenecks. This hypothesis is supported by a recent study which indicated that transition to metabolic inactivity occurs very rapidly following the onset of nitrogen limitation (Sanchuki et al., 2017). In general, cells suffer protein and DNA damage during a prolonged stationary phase, which is most likely caused by oxidation (Saint-Ruf et al., 2004, 2007). It is suspected that such damage and its repair may result in the induction of a lag phase following a long stationary phase, and that this effect may be a major cause of growth rate transition seen in this study.

### Identification of the Growth Modulon

As outlined, lag phases were induced by long-term stationary periods with different limiting conditions in preceding cultures. However, it has been shown that the response to previously limiting environments is condition-specific (Ishige et al., 2003; Silberbach et al., 2005). This aspect is also expressed in the heatmap of the dissimilarity matrix (Figure 2). Similarities between samples reflecting similar growth rates but different preceding conditions were markedly lower for the initial growth acceleration period. However, the faster the cells grew and the longer the preceding limitation period passed, the faster the differences diminished. Accordingly, samples were clustered with respect to growth rate as the primary influence followed by preceding conditions as a secondary effect. Furthermore, the influence of μ transition dominated the regulatory response, generally outcompeting the initial limitation period. For the purpose of excluding biased μ-modulon identification, only those DEGs exhibiting similar gene expression profiles irrespective of the preceding limitation period were analyzed (Figure 3).

### Pathway Enrichment Analysis

#### Central Carbon Metabolism

KEGG pathway enrichment analysis of gene clusters with similar expression profiles revealed that genes associated with central carbon metabolism were overrepresented in clusters 1 and 3. Especially, genes regulating glycolysis were found to exhibit a multifold increase of expression proportional to that of the growth rate. The same trend was true for the genes, *ptsG* (cg1537) and *ptsI* (cg2117), encoding the glucose uptake system as well as the gene encoding *iolT1* (cg0223) which has been shown to also enable glucose uptake. Genes *ppgk* (cg2091) and *cg2932*, which encode glucokinases were also in cluster 1. Interestingly, *gpmA* (cg0482) and *pgm* (cg2800) were the only genes encoding enyzmes of the glycolysis that did not amplify expression with increasing growth rates. No regulatory interactions were identified for these two genes. Apparently, their expression was decoupled from growth rate regulation. Several studies attempted to improve glycolytic fluxes through overexpression of single or multiple genes encoding proteins of glycolysis or sugar uptake systems (Müller et al., 1997; Ramos et al., 2004; Solem et al., 2010). A common observation was that sugar metabolism could only be improved slightly and that growth rates remained the same or decreased under aerobic conditions. Noteworthy, the tight growth link of glycolytic flux was no longer observed under oxygen deprived conditions without growth (Yamamoto et al., 2012; Tsuge et al., 2013). This stresses the inappropriateness of single engineering measures under aerobic conditions and hints toward requirement of concerted engineering of the total modulon. Therefore, basic regulatory regimes need to be engineered to improve fundamental traits such as sugar uptake or growth rates under aerobic conditions. Interestingly, this hypothesis may also be deduced from other independent findings. ALE experiments which yielded strains with increased maximum growth rates (Wang et al., 2018, ALE article submitted for publication) revealed that μ improvements could be mostly traced back to mutations in genes encoding transcriptional regulators of central carbon metabolism like GntR1 and RamA. Another ALE study outlined the reduction of pyruvate kinase (Pyk) activity as an important factor for growth and sugar uptake improvement (Pfeifer et al., 2017). However, the authors suggested that modulation of Pyk activity may lead to an increased phosphoenolpyruvate pool which in turn triggers a fundamental regulatory response, which is in line with our hypothesis.

This interesting response of genes encoding glycolytic enzymes and the glucose uptake systems raises the query whether the related PPP shows similar behavior. PPP is a major source of NADPH regeneration, thereby providing reducing equivalents for anabolism. In minimal medium, the rate of biosynthesis must be correlated to the growth rate to accommodate the rising demand for building blocks. Consequently, flux through the PPP is expected to increase with growth rate. However, such an increase was not reflected in transcript data and only *rpi* was part of the growth modulon. Accordingly, PPP flux is likely to be controlled at the metabolic level in contrast to glycolysis. This is in agreement with other studies. Moritz et al., 2000 reported that activity of 6-phosphogluconate dehydrogenase is feedback-regulated by its product NADPH. Additionally, it was shown that the PPP in *E. coli* has a flux capacity which was higher than was needed to sustain growth, which was most likely to allow rapid adaption to changes in NADPH demand (Christodoulou et al., 2018). Results from our *C. glutamicum* study may support the same hypothesis.

A substantial fraction of carbon funneled through glycolysis enters the TCA cycle which provides precursors of amino acid synthesis and reducing equivalents, such as NADH and FADH, serving as electron donors in the process of creating the transmembrane electrochemical proton gradient driving ATP generation. Accordingly, TCA cycle activities are expected to reflect cell growth and may follow gene expression profiles of glycolytic genes. But the differential expression patterns of glycolytic genes were not mirrored in the TCA cycle. In fact, the TCA cycle and the glyoxylate shunt contained only a few genes that are members of the μ-modulon. Thus, two trends are indicated: reactions leading to acetyl-CoA from acetate are upregulated at low growth rates; and *sucCD* (cg2037, cg 2038) and *actA* (cg2840) leading to increased succinate formation with rising μ. The first trend may mirror the cellular strategy which generates ATP via acetate formation even under non-growing conditions. The second may reflect the link between TCA based succinyl-CoA formation and its use via DapD, the 2,3,4,5-tetrahydropyridine-2,6-dicarboxylate *N*-succinyltransferase. DapD is part of the succinylase pathway representing one option for *C. glutamicum* to produce L-lysine from the intermediate tetrahydrodipicolinate. Because of its high abundance in protein compositions, cellular strategy may be to ensure growth coupled supply of succinyl-CoA for biomass synthesis via transcriptional regulation.

#### Iron and Metal Metabolism

Reduced iron (Fe^2+^) is an essential co-factor which ensures the proper functioning of many enzymes. Its uptake is strictly regulated since excess iron may be toxic, while low iron levels may hamper growth (Touati, 2000). In this study, genes associated with iron metabolism were strongly over-represented in cluster 2 and showed a sizable increase in expression during transition from μ = 0.2 to 0.4 h^-1^. Apparently, the cell reacted on a “perceived” iron shortage which was not present in the culture conditions. Furthermore, the transcriptional response did not cause a slowing of growth following phosphate or nitrogen limitation, but coincided with growth stalling following carbon limitation. This hints to a specific regulatory scenario independent of a sole iron shortage. Another factor influencing iron metabolism may be the presence of PCA in the medium. Liebl et al. (1989) demonstrated that PCA and other iron chelators improved growth performance of *C. glutamicum.* Because of this, PCA is suspected to be a facilitator of iron uptake in *C. glutamicum*. Notably, iron uptake mechanisms in *C. glutamicum* have not yet been clarified (Liebl et al., 1989; Dertz et al., 2006). Accordingly, PCA depletion may have triggered a transcriptional response. However, considering fast uptake kinetics (upt_PCA_ = 2.04 mmol gCDW^-1^ h^-1^, Unthan et al., 2014) where PCA concentration (c_PCA_) was 0.2 mmol L^-1^ and the initial biomass content was ∼1.2 g L^-1^, PCA levels were likely depleted in less than 1 h following inoculation, which was much faster than the appearance of the first transcriptional iron response. Consequently, other factors may have trigged the observed iron stress response at high growth rates. Disturbances within the intracellular iron storage system or oxygen stress may be a reason and further studies are needed to elucidate these details in *C. glutamicum*. In light of this result a follow-up study examining a growth rate transition after iron limitation might be interesting to gain further understanding of these mechanisms.

#### Other Growth Associated Functions

Interestingly, several important cellular functions are not part of the growth modulon even though the flux through related pathways is expected to increase with rising growth rates. For example, amino acid biosynthesis was not a part of the growth modulon. Either this indicates a very rapid gene expression dynamic that had already taken place during the initial lag phase, not yet monitored by sampling, or more importantly, it may mirror a fundamental cellular demand irrespective of growth conditions. Notably, amino acids are not only needed for growth-coupled biomass (protein) formation, but are also needed as building blocks for the protein fraction of ribosomes and for charging tRNAs, essential for all protein assembly. Both need cellular maintenance and require fast upshifts in case of suddenly improving nutrient supply to enable fast cell doubling. These arguments are in line with studies of Madar et al. (2013) who described the very early lag phase as a period of fast internal adaptation to the new environment. Following the initial amplification of biosynthetic pathways during the early phase after inoculation of fresh media, which is not covered within our data, we assume that biosynthetic fluxes are predominately controlled metabolically under the given experimental conditions.

The ribosome is another common growth associated cellular component. Studies have repeatedly demonstrated a linear correlation between growth rate and the amount of ribosomes in *E. coli*, at growth rates above 0.7 h^-1^. However, such a linear correlation was not evident within our transcriptional data. The reason for this is obvious in case of ribosomal RNA, since it was removed during the RNA-Seq sample preparation process, preventing further interpretation. However, analysis of genes encoding ribosomal proteins (rProtein) was possible. It was revealed that rProtein encoding genes were generally not part of the growth modulon under our experimental conditions. This is in agreement with the fact that formation of ribosomal proteins is mostly regulated at post-transcriptional level via translational feedback-regulation governed by rProtein abundance (Gourse et al., 1996; Nomura, 2014). Additionally, recent studies have indicated that the proportionality between ribosome numbers and growth rate is only valid for growth rates higher than 0.7 h^-1^ (Dai et al., 2016) and thus may not be evident during slower growth. As indicated by our data, it was postulated that a base amount of rProtein mRNA may be sufficient to account for increasing needs at low growth rates, and that translational feedback-regulation may successfully control rProtein demands.

### Identification of Active Regulons During Transition

Genes and regulons which are part of the μ-modulon were further studied via enrichment analysis (*p*_adj_ < 0.05). This approach was adopted to investigate the activity of known regulators and rank them according to statistical significance. We found maximum 64% genes of one regulon being part of the growth modulon (Table 1). Following the hypothesis that all genes of regulons serve the same common goal one might expect total coverage of gene expression. However, the partial involvement of regulons reflects the complex interaction of the regulatory network and the multifunctional role of the participating genes. Noteworthy, modulon identification followed an unbiased mathematical approach using filtering steps (e.g., a log_2_-fold change cutoff) combined with the comparison of three conditions. As such varying gene expression changes within a regulon were addressed individually. Intrinsically, the approach may have caused the rejection of genes with lowered expression changes and of outliers.

By using this approach, overrepresentation of iron metabolism associated genes in cluster 2 may be further specified as largely a part of the DtxR regulon. DtxR is the central regulator of iron metabolism in *C. glutamicum*. (Wennerhold and Bott, 2006). During growth rate transition, 64% of DtxR regulated genes exhibited a μ dependent regulation. This indicated that the DtxR regulon may be the most significantly enriched subset of genes. The intensity of the regulatory response showed the importance of iron for high growth rates in minimal medium, encouraging further investigation.

Among the other identified regulators, a group of carbon metabolism regulators, consisting of GlxR, SugR, RamA, and RamB (Shah et al., 2018), were found to play a dominant role in the regulation of central carbon metabolism during transition. In particular, SugR appeared to be a key regulator, as over 65% of its regulated genes were part of the growth modulon. With the exception of *ptsS*, all identified genes of the SugR regulon showed similar expression profiles and were members of cluster 1. This observation was interpreted as an additional indicator of the leading role of SugR for orchestrating crucial transcriptional changes during growth rate transition. However, many genes associated with carbon metabolism are members of up to four overrepresented regulons. This highlights the complex nature of the regulation of carbon metabolism and complicates clear identification of targets for strain improvement. However, regulators of carbon metabolism, such as GlxR, RamA, RamB, and especially SugR, may be considered major regulators of growth rate transitions and thus represent prime targets for increasing growth performance.

In terms of members, the SigA regulon is the largest growth regulon identified. The gene *sigA* (cg2092) encodes the housekeeping sigma factor *σ^A^*, the major sigma factor regulating gene expression during the exponential growth phase (Pátek and Nešvera, 2011). Whereas statistical significance of SigA regulon was the second highest, the total fraction of genes which belonged to the μ-modulon was comparatively low (25%). Most likely, this was due to the broad range of genes regulated by *sigA* (cg2092), which were targets of other regulators as well. Because of its complexity, SigA may not be the first target used to engineer *C. glutamicum* in order to achieve improved growth and sugar uptake rates. In fact, overexpression of *sigA* (cg2092) has been shown to induce increased by-product formation and a drop in specific growth rate (Taniguchi et al., 2017). The sigma factor, SigB, is a central part of the *C. glutamicum* stress response, replacing SigA during the stationary phase (Larisch et al., 2007). Its function causes interpretation of this sigma factor to be especially interesting in the context of changing growth rates. Unfortunately, no experimentally verified information is currently available on the SigB regulon. However, several genes have been predicted to be regulated by SigB, most of which are associated with carbon metabolism (Larisch et al., 2007; Ehira et al., 2008). Based on the predicted genes, the SigB regulon may have been identified as enriched during the transition, reflecting the decreased function of SigB during the exponential growth phase.

## Conclusion

The current study succeeded in identifying a growth modulon of *C. glutamicum* comprising 447 genes, which may be considered as the first, crucial step toward performing further metabolic engineering procedures to optimize growth rates and tightly linked biomass specific sugar uptake rates. Ten key regulator genes, representing a highly promising start for strain engineering, were found. However, no distinct regulators could be detected for 72% of the growth modulon genes, leaving room for future studies. Nevertheless, a key feature of growth regulation in *C. glutamicum* was found, which was the apparent tight link between the transcriptionally regulated glycolytic activity and the metabolically controlled pentose phosphate pathway, and to a large extent, TCA cycle activity. Furthermore, gene expression profiles of biosynthetic amino acid pathways did not show a significant dependence on growth either. This process may represent a strategy evolved by the original soil bacterium to ensure sufficient nitrogen supply by storing amino acids. In conclusion, these findings may help in increasing growth rates of *C. glutamicum* and fostering its use in industrial production processes further.

## Author Contributions

TH designed the study, carried out the experiments, analyzed the datasets, and drafted the manuscript. TB and JK measured the transcript samples, analyzed the datasets, and corrected the manuscript. MG and AN analyzed the datasets and corrected the manuscript. BB and RT conceptualized the whole study and corrected the manuscript. All authors read and approved the manuscript.

## Conflict of Interest Statement

The authors declare that the research was conducted in the absence of any commercial or financial relationships that could be construed as a potential conflict of interest.

## Footnotes

1 www.ebi.ac.uk/arrayexpress

2 www.coryneregnet.de
